# Supplementary material for: Dynamic metabolic exchange governs a marine algal-bacterial interaction
Source: eLife. 2016 Nov 18;5:e17473. doi: 10.7554/eLife.17473 (PMC5148602; doi:10.7554/eLife.17473)
Supplement: Supplementary file 1. — DOI: http://dx.doi.org/10.7554/eLife.17473.019 [file elife-17473-supp1.docx]

**Supplementary file 1.** Expression data ratios between algae in co-culture and in axenic culture.
